# N-acetylcysteine (NAC) and in psychodermatological conditions. Is it useful?

**DOI:** 10.1192/j.eurpsy.2023.450

**Published:** 2023-07-19

**Authors:** A. Jimenez, T. Gutiérrez, I. Rivera Ruiz, L. Montes, M. Reyes López

**Affiliations:** ^1^Psyquiatry; ^2^Dermatology, Hospital Universitario Reina Sofía, Cordoba, Spain

## Abstract

**Introduction:**

Pathologic grooming disorders can lead to clinically significant distress and functional impairment. Various psychopharmacological and nonpharmacological treatments have been used to ameliorate the symptoms of these disorders. N-Acetylcysteine (NAC) is a newer modality in the treatment of these disorders and has shown promise in treatment of obsessive-compulsive and related disorders.

**Objectives:**

To determine whether NAC is useful in the treatment of body-focused repetitive behavior disorders.

**Methods:**

A literature review was carried out in PubMed using the descriptors: “body-focused repetitive behaviour disorders” “grooming disorders” AND “N-Acetylcysteine”.

Six results were obtained when using the time limit of 5 years. We selected two of them for their relevance to the PICO question. Subsequently, the search was repeated using the same descriptors and the time limit in the Cochrane Library, Epistemokinos and Tripdatabase, in which no additional results were found.

**Results:**

Overall, thirty-three articles were included in these systematic reviews that we studied, which consisted of 23 case reports, one case series, and seven randomized controlled trials. Dosing of oral NAC ranged from 450 to 3,000 mg per day, and treatment periods lasted from 1 to 8 months. Side effects were uncommon, mild, and usually gastrointestinal in nature, with severe aggression reported in one child. Two randomized controlled trials showed a significant improvement in trichotillomania and excoriation disorder in adults. The other two were performed in pediatric population, showing no statistical difference. Two randomized controlled trials and six case reports studying the effects of NAC in patients suffering from trichotillomania (one performed in adults and the other one in infants) were included. Only the one performed in adults showed improvement when adding NAC to the treatment using the Massachusetts General Hospital, Hairpulling Scale (MGH-HPS). Four case reports and two randomized controlled trials included patients with excoriation disorder showed statistical differences in all of them. Three case reports and one randomized controlled trial in patients with onicotylomania showed a difference that wasn’t consistent after two months of treatment.

**Conclusions:**

There are multiple case reports an several clinical trials supporting both the safety and efficacy of NAC in the treatment of body-focused repetitive behavior disorders. Based on these positive preliminary results and the absence of serious adverse effects, carrying out a therapeutical trial with NAC is a plausible option in the management of this pathology, especially in those in which other therapeutic options have not been effective. Further studies are needed to develop a treatment algorithm and elucidate the difference in the efficacy of NAC between children and adults with this disorder.

**Disclosure of Interest:**

None Declared

